# Reading for Comprehension in Individuals with Down Syndrome, Autism Spectrum Disorder and Typical Development: Similar or Different Patterns of Ability?

**DOI:** 10.3390/brainsci11070828

**Published:** 2021-06-22

**Authors:** Maja Roch, Kate Cain, Christopher Jarrold

**Affiliations:** 1 Department of Developmental and Socialization Psychology, University of Padova, Via Venezia 8, 35131 Padova, Italy; 2 Department of Psychology, Lancaster University, Lancaster LA1 4YF, UK; k.cain@lancaster.ac.uk; 3 School of Psychological Science, University of Bristol, Bristol BS8 1TU, UK; c.jarrold@bristol.ac.uk

**Keywords:** down syndrome, autism spectrum disorder, simple view of reading, reading comprehension, homograph, decoding, language comprehension, reading for meaning

## Abstract

Reading for meaning is one of the most important activities in school and everyday life. The simple view of reading (SVR) has been used as a framework for studies of reading comprehension in individuals with Down syndrome (DS). These tend to show difficulties in reading comprehension despite better developed reading accuracy. Reading comprehension difficulties are influenced by poor oral language. These difficulties are common in individuals with DS and autism spectrum disorder (ASD), but they have never been compared directly. Moreover, the components of reading for comprehension have rarely been investigated in these populations: a better understanding of the nature of reading comprehension difficulties may inform both theory and practice. The aim of this study was to determine whether reading comprehension in the two populations is accounted for by the same component skills and to what extent the reading profile of the two atypical groups differs from that of typically developing children (TD). Fifteen individuals with DS (mean age = 22 years 4 months, SD = 5 years 2 months), 21 with ASD (mean age = 13 years 2 months, SD = 1 year 6 months), and 42 TD children (mean age = 8 years 1 month, SD = 7 months) participated and were assessed on measures of receptive vocabulary, text reading and listening comprehension, oral language comprehension, and reading accuracy. The results showed similar levels in word reading accuracy and in receptive vocabulary in all three groups. By contrast, individuals with DS and ASD showed poorer non-word reading and reading accuracy in context than TD children. Both atypical groups showed poorer listening and reading text comprehension compared to TD children. Reading for comprehension, investigated through a homograph reading accuracy task, showed a different pattern for individuals with DS with respect to the other two groups: they were less sensitive to meaning while reading. According to the SVR, the current results confirm that the two atypical groups have similar profiles that overlap with that of poor comprehenders in which poor oral language comprehension constrains reading for comprehension.

## 1. Introduction

According to an influential theoretical framework, the simple view of reading [1,2], both word recognition and listening comprehension are necessary for successful reading comprehension; each makes an independent contribution to reading comprehension, but neither is sufficient on its own. The simple view of reading provides a framework to identify the locus of reading comprehension difficulties and has been successfully applied to the study of reading in groups with atypical and typical neurodevelopment [3,4,5,6,7]. In this study, we used the simple view framework to explore and compare the reading profiles of individuals with Down syndrome (DS) and autism spectrum disorder (ASD) to those seen in a group of typically developing children (TD).

There is a growing body of research describing the reading profiles of individuals with a range of different neurodevelopmental disorders, and identifying the locus of their reading difficulties, with a particular focus on individuals with DS and ASD [8,9,10,11]. This has relevant theoretical and practical implications. Because oral language difficulties are associated with both DS and ASD, it is expected that these children experience difficulties with reading similar to those of children who are described in the literature as poor comprehenders: children who struggle with the understanding of printed texts but develop better than expected word reading and decoding skills [12]. However, just as in typically developing populations, there is heterogeneity, at least in the reading profiles of children with ASD; not all individuals with ASD have a poor comprehender profile. Further, for individuals with DS, the contribution of different word reading strategies to reading comprehension differs from typically developing readers [11]. With an increasing focus on the need to test the replicability of results to provide converging and accumulating evidence on a topic, we report new data on individuals with Down syndrome and those with autism spectrum disorder and contrast their performance with young readers with no known neurodevelopmental disorder. A particular innovation of our approach is the assessment of word reading in isolation and in context and an examination of reading for meaning using a homograph reading task. Reading for meaning is the main purpose of reading, and a better understanding of the strategies that support meaning construction is critical for developing appropriate reading instruction and remediation.

A growing number of individuals with DS have full access to formal education and acquire a range of literacy skills [13]. The following key findings emerge from the literature exploring their reading profiles. First, the reading comprehension of individuals with DS is predicted by both their word reading and their listening comprehension [14], confirming the validity of the simple view framework for understanding their reading outcomes. Second, individuals with DS typically present a “poor comprehender” profile: their listening comprehension is usually poorer than their word reading, such that the former makes a stronger contribution to their reading comprehension performance compared with typically developing readers [11,15,16,17]. Third, when compared with typically developing groups matched for reading comprehension, individuals with DS have relatively stronger word reading skills, indicating that language weaknesses are an important contributor to their poor reading comprehension [18]. Together, these studies support the view that individuals with DS have a poor comprehender profile.

For many years there was an assumption that reading skills represent an area of strength for individuals with ASD, at least in the area of word recognition [19]. For example, several studies demonstrate that individuals with ASD typically have age-appropriate or advanced sight word reading and non-word decoding skills [8]. However, group means can mask heterogeneity and some studies have revealed significant variability in this population [8]. Specifically, some individuals with ASD read accurately but show very poor comprehension, consistent with a hyperlexia reading profile, whereas others are poor at reading words and non-words, and some cannot decode non-words, despite a reasonable level of word reading skill. Recent work by McIntrye and colleagues has shown that reading comprehension in this population is a function of the severity of ASD symptoms: greater ASD symptomatology was related to poorer reading comprehension, and this relation was mediated by oral language skills [20,21,22].

The heterogeneity of the reading profiles of individuals with ASD was verified in a comprehensive study of 100 participants [10]: reading comprehension was an area of weakness for many, but not all, individuals, and word recognition skills were well developed for most participants. When groups of individuals with ASD with either good or poor structural language are compared, reading comprehension weaknesses have been found to be especially marked in those with poor oral language skills [23]. Thus, compared to individuals with DS, the reading profile of individuals with ASD is more heterogenous in nature with highly variable levels of achievement. However, consistent with the findings on DS, individual differences in reading comprehension in ASD are paralleled by differences in oral language skills [8].

To sum up, the profiles of DS and ASD reading skills show several commonalities, in particular: (a) both show relative strengths in word identification, in spite of a selective disadvantage in non-word reading [24,25,26,27,28]; (b) the relative advantage in word identification is not a guarantee that what is read is also understood for individuals with DS [11,18,29] and those with ASD [30,31,32,33].

Word reading accuracy tasks provide valuable information about the lexicon and retrieval of pronunciations, whilst responses to comprehension questions about a passage tell us about what has been understood. Neither tell us about the process of reading. One way to look at this relationship between what is read and what is understood is to use a task that measures how accurately homographs are read in different contexts. Homographs are word pairs that have the same orthography but differ in both their pronunciation and meaning (e.g., lead). The correct pronunciation of a homograph can only be determined by processing the meaning of the surrounding context. If the sentence has been understood correctly then participants should provide the contextually appropriate pronunciation of the homograph. Homograph reading has been investigated in some studies that involved individuals with ASD. These all consistently show that they tend to perform relatively poorly on the test, suggesting a failure of processing for meaning during reading [2,30,31,34,35,36,37]. Impaired homograph reading for this population has been interpreted as a deficit in context processing, within the theoretical account of “weak central coherence” [30] according to which individuals with ASD are less accurate in homograph reading because they process each word in isolation, ignoring the surrounding context.

To the best of our knowledge, homograph reading has not been investigated in individuals with DS, but there is some evidence that they process the meaning of the linguistic context at least to some extent in order to identify the meanings of unknown words [38], morphosyntatically complex sentences [39], and ambiguous sentences, such as idioms [11]. It was shown, however, that the ability to process the meaning of the context is a function of the ability to process a text. To better understand the reading strategies involved in completing the homograph-reading task, we investigated the relationship between homograph reading accuracy and the discrepancy between word reading and reading comprehension in both DS and ASD individuals and compared them to TD children.

## 2. The Current Study

To date, the examination of the reading profile of individuals with DS and ASD has not directly compared the profiles of the two groups. The purpose of the current study was to describe the reading profiles of individuals with DS and ASD and TD children and to identify the sources of individual differences in reading comprehension in the three groups. The evidence to date suggests that, whereas both atypical groups have reading comprehension difficulties, the source of the difficulty may differ: individuals with DS typically show strong word reading skills relative to listening comprehension; in contrast, individuals with ASD may also show this poor comprehender profile, but a sizeable number will likely show a different reading profile. In addition, we investigated the strategies employed for reading for meaning through an investigation of the accuracy of homograph reading.

The following research questions guided the study:

RQ1: Are there group differences in decoding, oral language comprehension, and reading comprehension? The three groups (DS, ASD, and TD) were compared on a large set of tasks measuring single word and non-word reading, receptive vocabulary, listening, and reading text comprehension in order to investigate group differences.

RQ2: To what extent is the relationship between reading comprehension and either decoding or language comprehension different across the three groups? Regression analyses examined to what extent each component predicts reading comprehension and whether a similar or different relationship holds for the three groups.

RQ3: To what extent do individuals with DS and ASD use context as they read homographs compared to TD children? The strategy of reading for meaning in the three groups was investigated through a homograph accuracy task. Two analyses were conducted to verify whether: (1) there is a facilitating effect of the context preceding the pronunciation of the homograph as an indicator that participants are reading for meaning; (2) there is a relationship between homograph accuracy and the other measures, particularly word and non-word reading accuracy and language comprehension (receptive vocabulary and listening text comprehension).

RQ4: To what extent do individuals with DS and ASD show a poor comprehender profile compared to the TD group? The heterogeneity of the poor comprehender profile was investigated in the three groups. The prevalence of a poor comprehender profile was established, and then, for each group, the accuracy with which reading skills, language comprehension, and reading for meaning predicted group membership was analysed.

## 3. Method

Three groups participated: 15 individuals with Down syndrome (mean age = 22 years and 4 months, SD = 5.6; 47% female), 21 individuals with ASD (mean age = 13 years and 3 months, SD = 1 year and 9 months; 19% female), and 42 typically developing children (mean age = 8 years and 1 month, SD = 1 year and 2 months; 58% female). The two atypical groups were selected from a larger sample on the basis of excluding any individual whose reading abilities were limited to letter recognition only. All participants had normal or corrected vision. To the best of our knowledge, there were no cases of comorbidity between DS and ASD among our participants. Participants with ASD were recruited from a school that specialised in the education of children with ASD. Participants with DS, instead, were contacted using a database of previous collaborations with the University of Bristol. Participants were recruited with their full consent (or parental consent where appropriate) and were paid an honorarium for their participation. Those under 18 received a book voucher, and those 18 and above received an equivalent payment, in thanks of their participation. Participants with TD were recruited in three different mainstream schools in Bristol and Lancaster. The Faculty of Science Human Research Ethics Committee (REC) at the University of Bristol approved the research on ASD (ref 453) and DS (ref 374). At Lancaster University, the Department of Psychology’s Ethics Committee approved the research on TD children in June 2009.

## 4. Materials and Procedure

All participants completed a range of standardised tests and experimental measures of word reading accuracy, reading comprehension, receptive vocabulary, and listening comprehension. The assessments were completed in a single session lasting 45 to 60 min. Tasks were administered to all participants in the same order.

### 4.1. Reading Comprehension 

Reading comprehension was assessed with the Neale Analysis of Reading Ability—Revised [40]. In this test, children read aloud a series of short stories and answer questions after each one. Some questions can be answered with reference to explicit details in the text, whereas other responses require the participant to generate an inference. The stories get progressively harder and we stopped testing when participants were not able to correctly answer any questions about the story. This procedure is different to the administration guidelines, in which the discontinuation rule is based on word reading errors only. Because of the significant number of poor comprehenders (with good word reading but poor text comprehension) in the DS and ASD populations, we implemented this discontinuation rule to minimise unnecessary frustration and distress to participants. We had no reason to believe that this adjustment produced any relevant differences in the results obtained. For consistency, we used both raw scores and age-equivalent scores. The comprehension raw score was obtained by summing the total number of questions answered correctly, which can then be transformed into a reading comprehension age according to the test norms. The NARA-II has a high test–retest reliability (>0.81) and a high internal reliability (>0.82).

### 4.2. Word Reading 

Three tasks assessed the accuracy of single word and non-word reading and word reading in context. Single word reading was assessed by presenting individuals with 20 irregular words of one or two syllables. In each case, stimuli were presented singly and sequentially on a computer screen in Times New Roman 80-point font, and the participant was asked to read and pronounce the item. One point was awarded for each correct response (score range: 0–20). Single non-word reading was assessed using the same method, with 20 non-words of one or two syllables: one point was awarded for each correct response (score range: 0–20). The two tasks were presented separately to minimise the likelihood that individuals would make lexicalisation errors for the non-words. These tasks have been used in previous work [26,41] and were demonstrated to be sensitive to individual differences in atypical populations.

Word reading in context was measured with the NARA-II [40], described above. The score for the number of words read correctly during the passage reading was recorded and transformed into reading age accuracy according to the test norms. The reliability of this test is 0.89.

Oral language comprehension. Receptive vocabulary and listening comprehension were assessed with separate tests. Receptive vocabulary knowledge was measured with the British Picture Vocabulary Scale (Third Edition) (BPVS; Dunn et al., 2009) [42] to provide a measure of breadth of vocabulary knowledge. Each participant is asked to select one of four pictures that best showed the meaning of a word spoken aloud by the experimenter. Raw scores consist of the number of correctly recognized words minus the errors. The test was administered and scored according to the guidelines in the manual: the standard scores have a mean of 100 (SD = 15). The split-half reliability of the task in our age range varies between 0.85 and 0.95.

Listening comprehension was assessed with Form 2 of the NARA-II. These were different passages to those used to assess reading comprehension and were read aloud to participants, with questions to tap comprehension asked after each passage. Testing stopped when the participants did not provide an answer to any of the story questions, following the same procedure as for the reading comprehension assessment. The number of correctly answered questions was transformed into a listening comprehension age. Because participants completed different numbers of stories and, therefore, different numbers of questions, we transformed raw scores into a listening comprehension age to ease comparison amongst participants and between groups. In addition, and for full transparency, we reported raw scores in addition to age-equivalent scores. The parallel form reliability of this test (across all relevant age groups) is 0.89.

Homograph reading task. Five homographs were used in the study (tear, row, bow, windy, and lead): the target stimuli were selected from previous studies on homograph reading [2,30,31,34,35,36,37]. We chose the target homographs that showed the best reliability in previous research and at the same time were sufficiently familiar to participants in our study (for both the meanings). The stimuli consisted of sentence pairs written with simple vocabulary and syntax in which the homograph was inserted; one sentence had the homograph with its more frequent pronunciation, the other with the less frequent pronunciation. Each sentence of the pair was preceded either by a meaningful context, which provided a clue for a correct pronunciation of the homograph, or a neutral context, which did not provide any clues to meaning (and, therefore, pronunciation). For stimuli preceded by the neutral context, the correct pronunciation of the homograph could only be deduced from the context following the homograph. Therefore, there were two independent manipulations: context—meaningful or neutral—and pronunciation—frequent or infrequent. This resulted in 20 items in total in four different conditions, only one showing reading for meaning and the other three serving as control conditions. For the purposes of the present study, we considered the responses in the condition in which the participants had to make use of the context in order to read the word accurately, that is, the one in which the pronunciation was infrequent and the context facilitating. An example of the four conditions of homograph reading is provided in Appendix A.

Each sentence was presented individually on a computer screen, with the item order randomized. Participants were asked to read aloud each sentence. Reading accuracy for each homograph was scored and any self-corrections were noted; these were anticipated in the neutral context condition if participants were reading for meaning. Thus, there were two scores: correct initial reading and correct reading after self-corrections had been taken into account. Each score had a maximum of 20 points. Internal consistency (Cronbach’s alpha) for the homograph task was moderate (α = 0.66). Finally, at the end of the task each participant’s knowledge of the meaning of the five homographs (ten meanings) was checked by asking them to point to the picture that best described the word’s meaning from a selection of four alternatives.

## 5. Results

### Reading, Oral Language Comprehension and Reading Comprehension: Group Comparisons

The data distributions for all measures were checked on the whole group. Except for the measure of vocabulary, which was found to be distributed normally, all the other variables did not match normality. However, for the TD group, all were found to be within a normal distribution, except for word and nonword reading accuracy. Separate between-subject ANOVAs were conducted for each variable using raw scores. Significant main effects were followed up with Tukey’s HSD post hoc test, and significant group differences are indicated in Table 1.

Age-equivalent scores are reported in Table 1 together with the raw scores in order to facilitate the reading and the interpretation of the results. Of note, the pattern of performance between groups was in line with previous research on DS (Laws et al., 2016) and ASD [14,18].

The three groups performed comparably in receptive vocabulary and single word reading; most of the individuals with DS, however, scored at the ceiling in the accuracy of single word reading. Both atypical groups had significantly poorer non-word reading and reading accuracy in context than the TD children. For reading comprehension and listening comprehension, both atypical groups obtained significantly lower comprehension scores than the typically developing controls. However, the group with ASD showed much greater variability in all measures compared to the DS group.

## 6. The SVR in the Three Groups: Pattern of Relations between Decoding, Language, and Reading Comprehension

**Figure 1:**
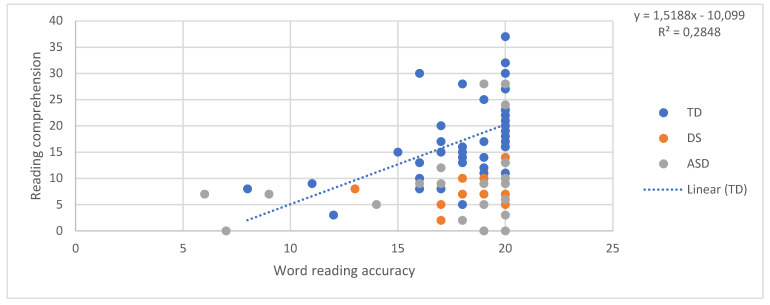
Word reading accuracy predicting reading comprehension.

**Figure 2:**
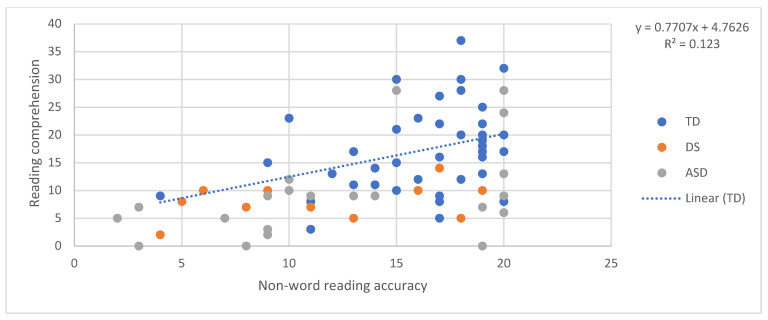
Non-word reading accuracy predicting reading comprehension.

**Figure 3:**
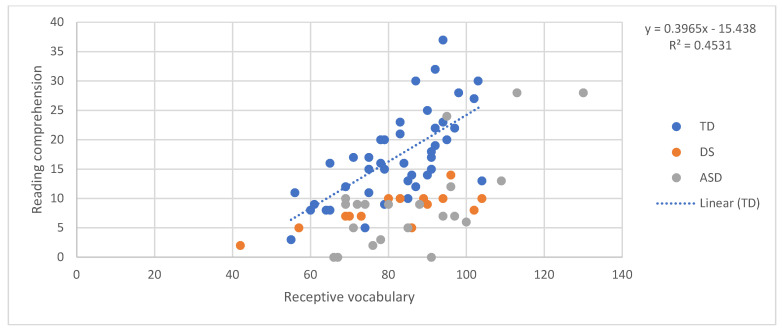
Receptive vocabulary predicting reading comprehension.

**Figure 4:**
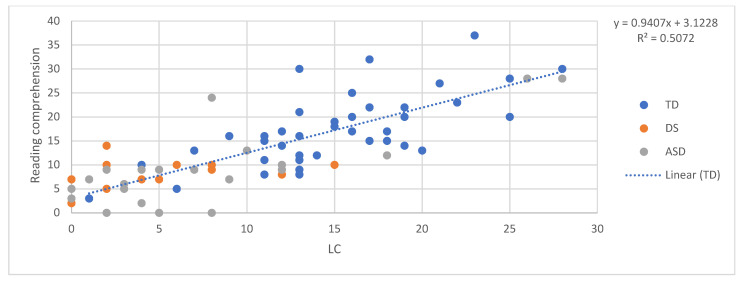
Listening comprehension predicting reading comprehension.

In order to examine the predictors of reading comprehension according to the SVR in the three groups, a relationship between the reading accuracy of words and non-words and reading comprehension, and between oral language skills (receptive vocabulary and listening comprehension) and reading comprehension, was tested. First, we ran four regressions, one for each component, predicting reading comprehension for the TD children. In other words, in each regression reading comprehension was the dependent variable and one of either word reading, nonword reading, listening comprehension, and vocabulary was the independent variable. Although the sample size of 42 typically developing children was arguably small for a regression analysis, the validity of the approach we adopted was shown by the fact that all of the regressions within this group, in Figure 1, Figure 2, Figure 3 and Figure 4, explain a meaningful amount of variance (minimum 12.3%, *p* = 0.02).

Then, the equation for the relationship between reading comprehension and each predictor in children with typical development was used to calculate expected reading comprehension scores for each individual with DS and each individual with ASD, given their score on the predictor variable in question. The “residual” discrepancy between the observed and the expected reading comprehension for each individual with DS and ASD was then standardised on the basis of the standard error in the estimate from the typically developing individuals’ regression [43]. The adequacy of the standardisation of the performance of the two atypical groups was not dependent on the size of these groups and was performed using the standard error in the estimate of the TD regression, and, therefore, it took into account the degree of confidence in this regression.

This produced a z-score for each participant that indicated whether their reading comprehension score was in line with what one would expect given their performance on the predictor variable in question. Negative z-scores indicate that participants had lower reading comprehension than expected given their performance on the target predictor. Figure 1, Figure 2, Figure 3 and Figure 4 provide a diagrammatic representation of these analyses. The average z-scores for each predictor are presented in Table 2.

All the z-scores, except the scores obtained with listening comprehension as the predictor, were significantly different from 0, that is, different from the performance of TD children. This means that both atypical groups scored less well on reading comprehension than would be expected given their vocabulary and word and non-word reading scores and that these scores are therefore less reliable in predicting their reading comprehension level. On the other hand, the fact that z-scores with listening comprehension as the predictor were not significantly different from zero suggests that a similar relationship between listening and reading comprehension might exist in the three groups, despite the fact that the two atypical groups show lower scores than TD children in both. Notably, the standard deviations and the figures indicate a higher variability among individuals with ASD in all the tasks. These results indicate that, for individuals with DS and ASD, reading comprehension is predicted by listening comprehension as we found for TD. For the atypical groups, both listening and reading comprehension were poor.

## 7. Reading for Meaning: A Homograph Task

The homograph reading task provides a window into how children process text as they read: processing the word in relation to the surrounding context is necessary for accurate pronunciation, otherwise the most frequent pronunciation will be retrieved. Knowledge of the two meanings of each homograph was high (*M* = 8.38, SD = 1.13 over 10 words) and did not differ significantly amongst groups: *F*(2,33) = 0.596, *p* = 0.557. The crucial condition in order to investigate the ability to read for meaning is the one in which a less frequent pronunciation is required but the context preceding the homograph provides a clue for the correct pronunciation. Performance in this condition was compared to performance when the context was neutral, e.g., the context did not provide any clue to identifying the correct pronunciation. Only the group with DS did not show a facilitating effect of the context. In contrast, the groups with ASD and with TD were more accurate in the facilitating context than in the neutral one. Table 3 shows the results.

In order to further investigate the strategy of reading ability for meaning construction, a subsequent analysis investigated whether the ability to read for meaning, that is, to accurately read the less frequent homograph in the facilitating context, was related to the other measures included in the study, namely, to word reading accuracy and comprehension measures. Table 4 shows these correlations.

For all groups, homograph reading accuracy was not significantly related to word reading accuracy, either for single item reading or for text reading. There was a single exception: a significant correlation between homograph and text reading accuracy in the group with ASD. For all three groups, homograph reading accuracy was significantly associated with measures of language comprehension: to listening comprehension in all three groups, to receptive vocabulary in the two atypical groups, and to reading comprehension in the ASD and TD groups.

## 8. A Poor Comprehender Profile in the Three Groups

As a next step, the reading profiles of each group were explored, specifically to determine the proportion of individuals with a poor reading comprehender profile in each. We used the definition of poor reading comprehension as a discrepancy higher than 6 months (or 1 SD) between text reading and reading comprehension ages (Cain & Oakhill, 2006) [44]. Using this criterion, we found a different proportion of poor comprehenders in each group: 12 out of 15 DS individuals (80%) were classified as poor comprehenders; 11 out of 21 (52.4%) individuals with ASD; and 11 out of 42 (26%) of the TD sample. The three groups of “poor comprehenders” showed a similar discrepancy between text reading and reading comprehension (*F*(2,33) = 1.13, *p* = 0.377): the mean discrepancies were 16 months for the DS group (SD = 7 months), 15 months for the ASD group (SD = 7 months), and 12 months for TD (SD = 6 months).

Finally, logistic regression was conducted to determine the accuracy with which word reading and listening comprehension scores predicted group membership. All participants with DS were correctly classified on the basis of these scores. This was not the case for the ASD and TD groups. For the ASD group, 8 out of 11 participants (72%) were correctly classified as poor comprehenders, whilst only 5 out of 10 (50%) were correctly classified as good comprehenders. For the TD group, 5 out of 14 participants (35%) were correctly classified as poor comprehenders and 26 out of 28 participants (92.2%) were correctly classified as good comprehenders. The addition of the homograph reading accuracy scores increased the accuracy of the classification of good comprehenders in both groups: the ASD group (70%) and the TD group (42%).

## 9. Discussion

The main aim of the current work was to further examine the reading comprehension and related skills of individuals with DS by analysing the ability to read for meaning within the theoretical framework of the SVR. The group of individuals with DS was compared to a group of individuals with ASD and to a group of children without neurodevelopmental disorders with comparable levels of single word reading accuracy and receptive vocabulary.

The rationale for comparing the two groups with atypical development is that both present a profile similar to that described in the literature as poor comprehenders but have never been compared directly. Our results provide further evidence for the validity of the SVR model in both typical and atypical populations by replicating the pattern of the results concerning single word and non-word reading, language comprehension, and reading comprehension in all three groups. However, a new important piece of evidence concerning the reading for meaning emerged by using a homograph task: individuals with DS seem less sensitive to meaning while reading compared to both ASD and TD children. Furthermore, almost all individuals with DS fell within the profile of poor comprehenders, whereas individuals with ASD showed a more heterogeneous profile, with only 50% of individuals matching the profile of poor comprehenders. The basic skills included in the SVR were sufficient to predict the reading comprehension profile of the individuals with DS, while the addition of the homograph task increased the accuracy of the prediction of poor vs. good reading comprehension in the groups of ASD and TD children. The results are discussed in the light of recent relevant literature organized in four relevant topics.

## 10. Word Reading, Oral Language Comprehension, and Reading Comprehension: Group Comparisons

Group comparisons revealed that the DS and ASD groups obtained comparable scores on measures of word reading but significantly weaker non-word reading than the typically developing controls. This pattern aligns with previous studies reporting poor decoding skills in individuals with DS [26,41] and ASD [29], which have been attributed to impaired phonological processing. Of note, real-world reading accuracy did not differ amongst the three groups.

Group comparisons further revealed that the DS and ASD groups obtained comparable listening comprehension and reading comprehension scores, again significantly weaker than those obtained by the typically developing controls. The weak text comprehension skills of the DS and ASD groups contrast with the comparable real-word reading performance of the TDs. These findings confirm previous literature on both English- and Italian-speaking individuals with DS [14,17,18] and English-speaking individuals with ASD [8,9,10]. These two languages differ for orthographic transparency, but the pattern of results is similar. Future studies should further address the generalizability of these findings by investigating other languages, for instance, logographic ones [45].

The current findings provide convergent evidence that the profile shown by both DS and ASD individuals is similar to that of poor comprehenders. This was further investigated by analysing the sources of individual differences in reading comprehension within the theoretical framework of the SVR.

## 11. The SVR in the Three Groups

Following the SVR, both reading accuracy and language comprehension were analysed as possible predictors of reading comprehension in all three groups. As predicted by the model, both factors, measured through multiple tasks, predicted reading comprehension in the TD group. On the other hand, the two atypical groups showed uneven predictions of reading comprehension. We used the equation for the relationship between each predictor (word and non-word reading accuracy, receptive vocabulary, and listening comprehension) of children with typical development in order to calculate expected reading comprehension for each individual with DS and with ASD. What emerged for both atypical groups is that only the level reached in listening comprehension predicted the level of reading comprehension, suggesting that listening comprehension is the most reliable predictor of reading comprehension for these groups. The relationships between the other factors and reading comprehension in DS and ASD differed from the relationship found in TD children. The individuals with DS and ASD showed lower reading comprehension than would be expected from their receptive vocabulary, word reading, and non-word reading. These results suggest that the two atypical groups have a profile that overlaps with that of (typically developing) poor comprehenders in which poor listening comprehension constrains reading comprehension abilities. This result is also consistent with previous work conducted with Italian-speaking individuals with DS and English-speaking participants with DS [14,18]. The results can also be related to studies conducted with individuals with ASD in which it has been shown that reading comprehension difficulties are related to levels of oral language skills [23]. In the current study, we did not take into account factors not considered within the SVR framework, which contribute to reading and reading comprehension skills as suggested by some previous works [46,47]. Future studies should include them in order to obtain a more complex and realistic picture of reading profiles in DS and ASD individuals.

The innovative aspect of the current work is that, for the first time, the two atypical groups were compared directly on the same set of tasks: this showed important commonalities in their reading profile. However, a further examination of the results revealed that the pattern of similarities in the profiles of DS to that of ASD were paralleled by some important differences.

## 12. Reading for Meaning: A Homograph Task

One of the most innovative aspects of the current work is related to the analysis of the ability to process meaning while reading, which was investigated through a homograph reading task. This task can be considered an implicit measure of comprehension monitoring since the correct and accurate reading of the homograph depends upon the processing of the meaning of the surrounding context. This task has been used in previous studies with individuals with ASD, but in this study it was adopted for the first time with individuals with DS. Individuals with DS tended to read with a more frequent pronunciation irrespective of the information provided by the context, showing low sensitivity to the information provided by the context. On the other hand, both individuals with ASD and TD tended to be more accurate in reading the homograph when the context provided a clue for its meaning. This finding differs at least in part from previous results. As far as individuals with ASD are concerned, it has been reported that they usually fail to use sentence context in pronouncing homographs and that this reflects weak central coherence [37,48]. However, there are two more recent studies that adopted more innovative paradigms to the study of homograph reading, showing that individuals with ASD do not show an absolute deficit in the ability to use contextual information. In fact, Hala et al. (2007) [49] showed that individuals with ASD were generally poor in reading homographs accurately but were able to use semantic primes to some extent in order to drive the correct pronunciation of the homograph. Further, Brock and Bzishvili (2013) [50] demonstrated through an eye-movement paradigm that individuals with ASD tended to increase their fixation times before pronouncing the homograph, suggesting that they adapted, at least to some extent, their reading strategy to minimize pronunciation errors. Although the current work adopted a classical paradigm for homograph reading, our results are more consistent with these findings. These data do not necessarily refute the central coherence account but rather highlight the possible variability within the group of individuals with ASD in their sensitivity to the context. Our results suggest that this sensitivity might be related to the extent to which individuals with ASD develop the ability to read for meaning. In other words, it looks like at least some individuals might be able to process meaning while reading.

As far as individuals with DS are concerned, previous work has shown that they, in contrast, tend to show greater sensitivity to the context and are helped by a short context in order to build meanings of unknown words [38], morphosyntactically complex sentences [39], and ambiguous sentences [11]. However, the current data suggest that they are less likely to adapt their pronunciation of the homograph according to the context. Three tentative explanations are provided for the discrepancy of the current and previous findings just mentioned. First, in previous studies the ability to use context was measured through tasks targeting the recognition of meaning, whereas in the current study it was measured through a reading accuracy task. Second, whereas previous studies measured the ability to use context off-line and asked about meaning explicitly, in the current work, the processing of meaning was measured implicitly, and this measurement occurred on-line. Third, in previous work the tasks were presented orally, whereas here the task enabled us to measure participants’ ability to process the meaning while reading. It is possible that individuals with DS are indeed able to process to a certain extent the meaning of a short context, but they are enabled to do so if they have to read contemporaneously. In other words, we speculate that reading skills and comprehension of what is read might be even more dissociated in this population. All three reasons can explain these apparently contradictory results. As already noted, this is a small-scale exploratory study that adopted this task for studying reading for meaning in individuals with DS for the first time. This task has considerable potential for studying comprehension strategies used during the process of reading. It would be important to further investigate the variation in the sensitivity to the context and, in particular, to focus on how individuals with DS build meaning while reading and after they have read a text. Moreover, future studies should also take into account cultural settings and linguistic features of the language in which participants are tested, since these characteristics may influence how reading developed, as demonstrated in studies conducted in a logographic language such as Chinese [51].

Finally, for all three groups, an association was found between reading for meaning (the homograph task) and oral language skills, a finding that highlights the importance of oral language skills for the development of reading and reading-related skills. On the other hand, we failed to find any reliable correlation between homograph reading accuracy and the measures of reading accuracy (both word and non-word reading). This further confirms that homograph reading is not related to the recognition of the single word but rather to the search of meaning in context. The analysis of reading for meaning therefore revealed some important differences between individuals with DS and the other two groups. This was further highlighted by the analyses of the poor and good comprehender profiles.

## 13. A Poor Comprehender Profile in the Three Groups

Within the group of individuals with DS, the number of participants who showed a relevant discrepancy between text reading accuracy and comprehension levels (i.e., >6 months) and could be defined as “poor comprehenders” was significantly higher than in the other two groups: it was 80% for individuals with DS, 51% for ASD, and 26% for TD. Notably, nobody within the two atypical groups showed a profile characterized by an opposite discrepancy, namely, in which word reading lagged behind text comprehension. In a previous study conducted by Nash and Heath (2011) [18], a similar number of DS participants were classified as poor comprehenders. No such estimates are reported for individuals with ASD, although Nation et al. (2006) [8] reported that 65% percent of participants with ASD showed poor reading comprehension, namely, having standard scores at least 1 SD below population norms, and 38% scored more than 2 SD below population norms. Although the data are consistent with the previous literature, we should point out that in this study we used a somewhat different procedure for the administration of the NARA (Neale, 1997) [40]. This may have affected the results, but we believe our procedure was appropriately adapted for the assessment of all children and young people who read and decode but have difficulty understanding the meaning of what they are reading.

All participants with DS were correctly classified as poor comprehenders using word reading and listening comprehension as predictors. These skills are accurate predictors of poor comprehension in ASD and of good comprehension in the TD group. By the addition of the homograph task as a predictor, the accuracy of the prediction of group membership increased significantly. This finding once again suggests that the homograph task, appropriately tested for validity in future studies with a larger number of participants, could be a relevant tool for analysing reading comprehension strategies.

Therefore, we conclude that both DS and ASD individuals tend to show a profile similar to (typically developing) poor comprehenders but that the sources of reading comprehension failure might differ for the two atypical groups. Cain and Oakhill (2006) [44] found that there is no single underlying source of poor reading comprehension, but rather, different cognitive and linguistic skills play a role in determining the pattern of a persistent poor comprehension profile. As a consequence, it is insufficient to define individuals according to the profile of poor comprehenders on the basis of the level reached in target abilities since group comparisons may obscure crucial differences in the underlying profile. This substantial heterogeneity is particularly relevant in informing both theoretical models of reading development and educational practice. For instance, in order to reduce the existing gap between text word reading and comprehension, different intervention strategies might be effective for the three groups considered in the current work. The current study provides an important step further in our understanding of the processes underlying reading comprehension by using a homograph task. We demonstrated that measuring reading for meaning is important for the prediction of good and poor reading comprehenders with ASD and TD. In contrast, we can accurately predict the reading profile of DS without this measure. Again, we stress the tentative speculative interpretation that individuals with DS have relevant difficulties in processing the meaning of a text while they are reading it.

## 14. Conclusions

The current work provides new evidence regarding the reading profiles of individuals with DS by comparing them both to individuals with ASD and to TD children. This is a small-scale study and represents a starting point for further research investigating reading for meaning in atypical populations. Both individuals with DS and ASD show atypical development of different component skills of reading. These similarities in the profile are paralleled by important differences. The pattern of findings demonstrates that the precise balance of reading skill strength and weakness in children identified as poor comprehenders is heterogeneous such that the identification of poor comprehension is not sufficient for a complete and adequate description of an individual’s difficulties, since the source of the poor comprehension may differ both across and within each population.

As far as similarities are concerned, both ASD and DS individuals show relatively well-developed reading skills: this might be due to the intrinsic characteristics of the two conditions but could also be related, at least in part, to the interventions usually delivered to these people. Both in clinical practice and in educational settings, interventions related to literacy skills are commonly focused on reading skills and are less likely to be oriented to comprehension and its components. The current study, in line with various previous studies, provided further evidence for a substantial influence of oral language in determining individual differences in reading comprehension. This reinforces the idea that the target of intervention should be moved from reading to comprehension in general and to oral language skills in particular.

Another relevant focus for future studies should be a deeper investigation of the strategies adopted to construct meaning from text, strategies that might vary substantially in different populations having an apparently similar profile. In particular, individuals with DS appear to be more impaired in the ability to engage in simultaneous decoding and reading for meaning.

Finally, further effort should be made to define in a more comprehensive way the poor comprehender profile. Convergent findings provide evidence that defining a category of poor comprehenders is reductive and tends to obscure the heterogeneity of individual differences: as in the current study, apparently similar profiles might hide differences in the underlying causes of poor reading comprehension.

## Figures and Tables

**Table 1:** Descriptive statistics for all measures (raw and standard scores are reported where available).

	DS (*N* = 15)	ASD (*N* = 21)	TD (*N* = 42)	ANOVA
Single word reading (max = 20)	18.34 (1.91)	17.12 (4.40)	17.90 (2.74)	*F*(2,77) = 0.73, *p* = 0.448
	13–20	6–20	8–20	
Single non–word reading (max = 20)	12.13 (5.21)	12.4 (6.19)	16.08 (3.41)	*F*(2,77) = 6.52, *p* < 0.01 DS = ASD < TD
	4–20	2–20	4–20	
Text reading: raw scores	37.13 (37.13)	31.48 (17.27)	48.31 (8.39)	*F*(2,77) = 11.65, *p* < 0.001 DS & ASD < TD
	21–50	(0–63)	(30–69)	
Text reading: age equivalent	89.32 (9.67)	84.67 (17.90)	97.82 (12.11)	
	73–105	54–121	68–126	
Receptive vocabulary: BPVS raw scores	81.23 (15.63)	86.67 (17.15)	86.73 (10.95)	*F*(2,77) = 0.85, *p* = 0.429
	47–110	66–130	64–104	
BPVS standard scores (M = 100, DS = 15)	80.32 (17.25)	106.78 (27.89)	91.90 (16.94)	
	43–106	78–144	55–120	
Listening comprehension: raw scores	5.40 (4.67)	7.95 (7.78)	14.93 (5.75)	*F*(2,77) = 17.11, *p* < 0.001 DS & ASD < TD
	0–15	0–28	1–28	
Listening comprehension: age equivalent	67.52 (13.08)	76.9 (23.95)	94.22 (14.76)	
	48–96	54–141	60–130	
Reading comprehension: raw scores	8.2 (2.86)	9.29 (8.21)	17.17 (7.59)	*F*(2,77) = 13.29, *p* < 0.001 DS & ASD < TD
	2–14	0–28	3–37	
Reading comprehension: age equivalent	76.31 (7.41)	79.85 (23.95)	99.30 (18.93)	
	61–92	54–136	63–154	

**Table 2:** Mean z-scores (standard deviations) for each predictor in the two atypical groups.

Predictors	DS	ASD
Word reading	−1.469 (0.549)	−1.367 (1.307)
Non-word reading	−0.814 (0.557)	−0.695 (1.088)
Listening comprehension	−0.001 (0.794)	−0.431 (0.714)
Vocabulary	−1.433 (0.893)	−1.603 (0.875)

**Table 3:** Mean accuracy for homographs with less frequent pronunciation according to the context manipulation.

	Facilitating Context	Neutral Context	*t*-Test
DS	1.81 (1.29)	1.40 (1.22)	*t*(14) = 1.58, ns
ASD	1.51 (0.98)	0.71 (0.72)	*t*(20) = 3.63, *p* < 0.01
TD	1.71 (1.33)	0.81 (0.94)	*t*(41) = 3.91, *p* < 0.001

**Table 4:** Correlations between homograph reading accuracy, reading accuracy, and language comprehension.

		Reading Accuracy Measures			Language Comprehension Measures		
		Words	Non-Words	Text	Reading Comprehension	Listening Comprehension	Vocabulary
Reading for meaning	DS	−0.191	0.201	0.228	0.292	0.515 *	0.615 *
	ASD	0.225	0.615 *	0.415	0.608 *	0.536 *	0.778 *
	TD	0.361	0.088	0.348	0.498 *	0.417 *	0.282

* *p* < 0.003 (corrected for multiple comparisons).

## Data Availability

The data presented in this study are available on request from the corresponding author. The data are not publicly available due to ethical reasons.

## Appendix A

The homograph task stimuli example across the four conditions.

### Appendix A.1. Facilitating Context; Frequent Pronunciation

The show in the theatre ended. The actors took a bow while everyone clapped.

### Appendix A.2. Neutral Context; Infrequent Pronunciation

The room was full. The man gave a bow at the end of his speech.

### Appendix A.3. Facilitating Context; Infrequent Pronunciation (Target Condition in Which Reading for Meaning Is Required)

I sat down to tie my shoelaces. First, I made a bow, and then I tied a knot.

### Appendix A.4. Neutral Context; Infrequent Pronunciation

Julie was very happy. She made a bow to tie in her hair.
